# Predictability of Maxillary Molar Distalization and Derotation with Clear Aligners: A Prospective Study

**DOI:** 10.3390/ijerph20042941

**Published:** 2023-02-08

**Authors:** Vincenzo D’Antò, Rosa Valletta, Roberto Ferretti, Rosaria Bucci, Robertas Kirlis, Roberto Rongo

**Affiliations:** 1 School of Orthodontics, Department of Neurosciences, Reproductive Sciences and Oral Sciences, University of Naples Federico II, Via Pansini, 5, 80131 Naples, Italy; 2 Private Practice VIC Clinic, 03162 Vilnius, Lithuania

**Keywords:** aligners, movement accuracy, distalization, orthodontics, three-dimensional evaluation

## Abstract

Clear aligners are employed daily for the treatment of Class II malocclusions, when a valid option involves distalization and derotation of the upper first and second molars. Evidence regarding the predictability of these movements is slight, and the treatment outcome that clinicians plan may not be achieved. Therefore, the purpose of this study is to assess the accuracy of distalization and derotation with clear aligners. Geomagic Control X, a 3D quality control software, was used to superimpose digital models of the pre-treatment, post-treatment, and virtual plan (ideal post-treatment) measurements of 16 patients (4 M, 12 F; mean age 25.7 ± 8.8 years). Linear and angular measurement tools were used to calculate the amount of tooth movement prescribed and achieved. Distal displacement of the buccal cusps had an overall accuracy of 69% for the first molar and 75% for the second molar. The accuracy of molar derotation was higher for the first molar (77.5%) than for the second molar (62.7%). The aligners were not able to achieve 100% of the ideal post-treatment result; thus, planning of refinements is often needed. However, clear aligners can be regarded as a valuable option for the distalization of first and second molars.

## 1. Introduction

Clear Aligner Treatment (CAT) has brought new possibilities into the orthodontic world by reducing the discomfort associated with traditional orthodontic treatment and providing both patients and clinicians with a highly aesthetic treatment solution [1,2]. Aligners not only present better aesthetic performance compared to fixed aesthetic labial appliances [3], but also allow the patient to maintain better oral hygiene [4], as well as presenting good mechanical and chemical properties [5]. Nevertheless, the low predictability of dental movements seems to be a limitation, and it changes both with the type of prescribed movement and with the use of auxiliaries, such as attachments or pressure points [6,7]. Pure tipping, which is when the center of rotation is localized between the center of resistance and the tooth’s apex, is the most predictable movement, whereas any movement requesting radicular control often shows a predictability lower than 50% [8].

CAT was initially dedicated to the resolution of mild to moderate crowding or minor space closure [9]. With the introduction of auxiliaries, the evolution of materials and the growing interest of patients in the opportunity for aesthetic treatments, clinicians have tried to employ this system to deal with more and more complex malocclusions. Therefore, aligners have been used to treat sagittal malocclusions, vertical malocclusions, and transversal malocclusions, and have even been utilized in extraction cases [10,11,12,13].

When it comes to the correction of a dentoskeletal Class II relationship, the first option is functional orthopedic treatment, which is limited to the period of growth and to the skeletal component [14,15]. A valid alternative is maxillary molar distalization, a treatment mechanic useful to achieve a Class I canine relationship, gain space, and retract the upper anterior teeth. Both extraoral and fixed intraoral appliances can be used in conventional orthodontic therapy to distalize upper molars. Headgear is an effective device that exploits extraoral anchorage, without unwanted effects on other teeth, but it must be worn from 12 to 14 h a day, and patient compliance is often poor [16]. Intraoral fixed appliances, such as the pendulum appliance and distal jet [17,18], were introduced as more aesthetically-acceptable alternatives to headgear, since they minimize or eliminate the need for compliance; by using the palate, teeth, or both as sources of anchorage, the main side effect is anterior anchorage loss [19]. Moreover, intermaxillary Class II elastics and other intraoral fixed appliances (Herbst, Forsus) could be used to improve a molar Class II relationship in children and adolescents, but the main outcomes involve dental advancement of the mandibular arch [20,21,22]. 

CAT is considered to be a valid alternative to traditional orthodontic systems for distalizing upper molars; sequential distalization protocol starts with the second molar moving distally, and only when it reaches 50% of the planned movement does the first molar start moving, and so on up to the canine. En masse anterior retraction represents the last phase of the treatment protocol, and attachments from the canine to the second molar are used to maximize posterior anchorage. In addition, inter-arch Class II elastics are frequently used to minimize the risk of anterior anchorage loss. Nevertheless, by applying forces at the clinical crown level, and not at the center of resistance, bodily mesio-distal movement is difficult to realize, and tipping is often the unwanted result [23]. In order to improve predictability, attachments are used to create a moment, counteracting dental tipping, although some degree of tipping may occur [24,25].

Aligners appear to be effective in controlling bodily distalization of maxillary molars of 1.5 mm without any significant vertical or mesio-distal tipping movements [26,27]. Simon et al. [28] reported an accuracy of 87% when a mean distalization of 2.7 mm was prescribed, supporting the high predictability of distalization and remarking on the importance of staging as an essential aspect of treatment planning. Saif et al. [29] reported a lower accuracy of 73.8% when 2.6 mm mean distalization as prescribed, and a slightly higher accuracy of the maxillary first molar (75.5%) over the second molar (72.2%).

Molar derotation is also necessary during the correction of a Class II molar relationship. According to the literature, tooth rotation is difficult to achieve, particularly when it comes to conical-shaped teeth such as canines or premolars; attachments and IPR may improve predictability [24,30].

The mismatch between the digital setup and the actual result is a real issue that affects clinical practice, and the literature regarding CAT predictability is unable to provide unambiguous responses, with overall dental movement predictability varying between 55% and 72% [31]. A possible explanation is that, unlike with Ni–Ti arches, stress relaxation (the force that the aligner releases over time) decays exponentially in the very first hours [8,32]. Furthermore, the polymeric molecular structure undergoes intraoral degradation, causing a reduction of the forces delivered during the treatment [33,34,35]. Knowing the limitations of this treatment could empower clinicians to make the most of the system’s potential, for example, by planning more aligners with less movement built into each one [8].

In addition, most of the data reported in the literature is based on the Invisalign system, while many other competitors on the market use different materials, different thicknesses, and different planning software, which may affect the biomechanics and predictability of dental movements.

Therefore, the objective of this study is to evaluate the predictability of maxillary molar distalization and derotation using clear aligners.

## 2. Materials and Methods

The study protocol complied fully with the principles of the Helsinki Declaration and was approved by the Ethics Committee of the University Federico II (352/21).

### 2.1. Inclusion/Exclusion Criteria

Sixteen patients (4 males, 12 females; mean age 25.7 years, standard deviation 8.8, range 18–45.5 years old) were selected according to the following inclusion criteria: adults with no previous orthodontic treatment; prescription of maxillary molar distalization and derotation; non-extractive treatment, except for maxillary third molars; absence or previous extraction of maxillary third molars; and no combined treatment with fixed oral appliances or Temporary Anchorage Devices. Patients with syndromes [36], cleft palate [37,38], and local or systemic conditions or pharmacological treatments that could affect tooth movement [39] were excluded from the study. To account for model superimposition error, and because of irrelevant clinical significance, prescription values below cutoffs of 0.5 mm for distal displacement and 2° for derotation were excluded from the study.

### 2.2. Treatment Protocol

**Figure 1:**
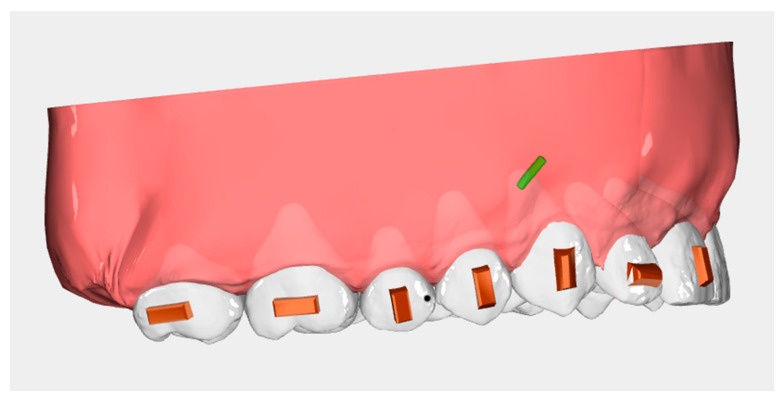
Attachment protocol: horizontal rectangular attachments on first and second molar.

All patients were treated with Ordoline aligners (UAB Ordoline, Vilnius, Lithuania). The attachment treatment protocol included horizontal rectangular attachments [40] on both first and second molars (Figure 1). Patients were instructed to wear the aligners for at least 22 h a day, only removing them during meals and oral hygiene procedures. All cases were treated with a distalization staging protocol of 50%, and patients were instructed to wear the elastics all day. Staging protocol of 50% is characterized by a sequential movement of teeth, where one tooth starts moving after the previous tooth achieves 50% of the prescribed movement, i.e., the first molar starts to distalize when the second molar reaches 50% of its distalization. Upper third molars were always extracted when present, at least 14 days before the delivery of the aligners. Aligners were changed every 10 days.

### 2.3. Data Collection

For each patient, three digital dental models (STL files) of the maxillary arch were gathered: pre-treatment (T0), the virtual plan at the end of the first set of aligners (T1), and the post-treatment digital model at the end of the distalization phase (T2). The pre-treatment and post-treatment digital models were acquired by means of an intraoral scanner (IOS, Trios 3, 3Shape, Copenhagen, Denmark). The virtual plan STL file was exported to investigate the predictability of the prescribed movements.

### 2.4. Superimposition and Measurement Protocol

The digital dental models were imported into Geomagic Control X (3D Systems, Rock Hill, SC, USA), a 3D metrology software that superimposes STL files and makes both linear and angular measurements. T0 and T1 were initially compared to establish the amount of movement prescribed. T0 was assigned to “reference data” and T1 to “measured data”; the teeth were then segmented in T0.

The buccal cusp tips of the first and second molars were identified by an operator and used as landmarks to measure the amount of distalization. In order to measure the actual displacement of the same point, each molar was sequentially chosen as a reference for a surface-based best fit, after which a landmark was identified at the same time in both T0 and T1. To measure derotation, a vector passing through the disto-buccal cusp and mesio-palatal cusp was created for each molar.

For global alignment, the method adopted was similar to the one reported by Grunheid et al. [41]. An initial 3-point alignment, based on the mesio-buccal cusp tips of the first molars and the mesial-incisal point of the right central incisor, was then refined with a global best-fit registration with 50 iteration counts. The points used for the initial alignment were also used to define the occlusal reference plane. A coordinate reference system where the XY plane is the transversal plane, the XZ plane is the sagittal plane, and the YZ plane is the coronal plane was created.

**Figure 2:**
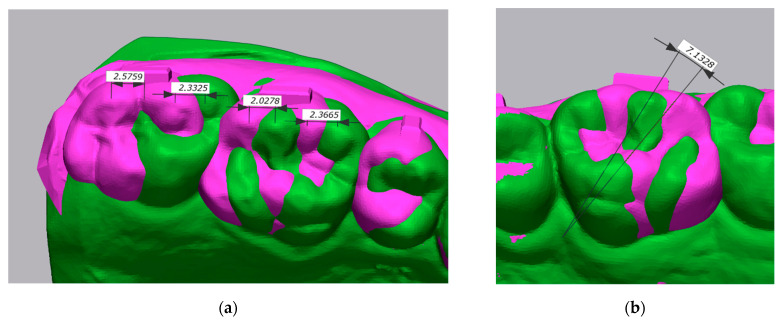
(**a**) Distal displacement of the buccal cusps measured on the sagittal axis: T0 in green; T1 in purple. (**b**) Molar derotation measured on the occlusal reference plane: T0 in green; T1 in purple.

Distal displacement of the buccal cusps was measured on the sagittal axis. The angle between each vector in T0 and its correspondent in T1 was measured on the occlusal reference plane (Figure 2).

The T2 STL file was imported into Geomagic in place of T1 and compared with T0 in order to determine the Achieved Movement. Sequential surface-based best fits of the occlusal surface of each molar allowed the operator to accurately identify the same landmarks. Global alignment, distal displacement of the buccal cusps, and derotation measurements were carried out as described above.

### 2.5. Prescription, Achieved Movement, and Accuracy

Distal displacement of the mesio-buccal (MB) cusp, distal displacement of the disto-buccal (DB) cusp, and derotation were analyzed for each molar according to the following variables:

- Prescription was the amount of distal displacement or derotation measured when comparing pre-treatment (T0) and virtual plan digital models (T1).
- Achieved Movement was the amount of distal displacement or derotation measured when comparing pre-treatment (T0) and post-treatment digital models (T2).
- Accuracy, expressed as percentage of achieved vs. planned, was calculated as follows:

Accuracy = 100 − [(Prescription − Achieved Movement)/Prescription] × 100

Finally, Overall Accuracy was calculated as the mean of the Accuracy of both buccal cusp tips for each molar.

### 2.6. Statistical Analysis

Considering upper molar distalization as the main outcome, an effect size of 0.6 was calculated by a previous study [29]. A sample size of 24 first molars and 24 second molars was needed, using a paired *t* test with an alpha error of 0.05, to achieve 80% power.

Intra-examiner and inter-examiner reproducibility of the measurements were evaluated by means of the Intraclass Correlation Coefficient: 20% of the digital dental models were re-analyzed by the same operator and again by a different operator 4 weeks after the first examination. The statistical package SPSS (IBM, Chicago, IL, USA) was used.

Descriptive statistical analysis included means, standard deviations, and C.I. 95% of Prescription, Achieved Movement, and Accuracy. A Shapiro–Wilk normality test was performed to assess the distribution of the data. A Student’s paired *t* test and Wilcoxon signed-rank test were used to evaluate whether differences between Prescription and Achieved Movement were statistically significant. A *t* test for unpaired data was used to compare Accuracy between the first and second molars. The significance level was set at 0.05.

## 3. Results

### 3.1. Intraclass Correlation Coefficient

The ICC was used to assess the reproducibility of measurements made by different examiners, or by the same examiner at different moments; values below 0.500 indicate poor agreement, whereas values above 0.900 indicate excellent agreement. The ICCs for intra-examiner and inter-examiner were, respectively, 0.995 and 0.986.

### 3.2. Prescription vs. Achieved Movement

Table 1 shows the means, standard deviations, upper limits, and lower limits, with 95% confidence intervals, of both Prescription and Achieved Movement. In every subgroup a statistically significant difference between Prescription and Achieved Movement was found. The overall mean distal displacement Prescription was about 2 mm, whereas the mean Achieved Movement was about 1.5 mm. Regarding derotation, 11.6° was the mean Prescription and 7.2° was the mean Achieved Movement.

### 3.3. Accuracy

Table 2 shows the mean, standard deviations, and 95% CI of Accuracy. No statistically significant difference was found between the Accuracy of each subgroup and its correspondent when comparing first and second molars. The highest Accuracy of distal displacement was reported for the mesio-buccal cusp of the second molar (79.9%); the lowest Accuracy was reported for the mesio-buccal cusp of the first molar (68%). The Overall Accuracy was 69.4% for the first molar and 75.2% for the second molar. Derotation was more accurate for the first molar (77.5%) than for the second molar (62.7%).

## 4. Discussion

With the objective of evaluating the accuracy of the distalization and derotation of upper molars, this study was designed to focus on the first phase of the treatment, and measurements were taken after the end of the distalization movement. The results show that distalization of the buccal cusps of maxillary molars is effective, and the accuracy varies between 68.0% of the mesio-buccal cusp of the first molar and 79.9% of the mesio-buccal cusp of the second molar. 

Studies performed with the Invisalign^®^ aligner system present comparable results. Simon et al. [28], in 2014, reported an accuracy of 87% for the distalization of upper molars; their sample was composed of 15 patients, of which 8 had attachments and 7 did not have any attachment. No statistically significant difference was found between the Prescription and Achieved Movement groups, nor between the group with and the group without attachments. Despite lacking evaluation of molar tipping movement, in the conclusion of their study it was reported that bodily tooth movements, including distalization, can be accomplished with aligners. Rossini et al. [42] considered this study of methodologically limited quality; therefore, those results should be regarded with caution.

A recent study from Saif et al. [29] reported an accuracy of 73.8% based on a larger sample that included 142 maxillary molars. Aligners were considered effective when 2 to 3 mm of molar distalization were achieved. In this study, no standardized protocol of attachments was adopted, and only 56.3% of the molars had an attachment. This study also did not show any statistically significant difference between the group with and the group without attachments. Differences in results between our study and the studies of Simon et al. and Saif et al. were mainly due to the difference in the measurement of distalization. Indeed, they measured the overall distal movement of the molars on the sagittal axis, whereas our study was specifically designed to investigate the distal displacement of the buccal cusps. Furthermore, the use of Class II elastics may play a role in the variation of results among studies. In our study, patients were asked to wear elastics bilaterally for 24 h of the day. Often, in clinical settings, Class II elastics are used as anchorage reinforcement and to improve the distalization achieved by the aligners, [21,22] but it is difficult to evaluate how much distalization is due to the aligner and how much is due to the elastics.

Geomagic Control X is a professional 3D quality control software that can perform both surface-based digital model superimpositions and linear and angular measurements [43]. Digital model superimposition, ideally, requires stable anatomic landmarks which are not modified by growth or bone remodeling following orthodontic treatment; nevertheless, literature shows no consensus regarding the best technique for reliable superimpositions [44]. As far as the maxillary arch is concerned, numerous studies identify the palate as a potentially reliable area for superimposition, and in particular the area that includes the medial two-thirds of the third rugae and the region 5 mm dorsal to them, but these studies are very heterogenous and show a high risk of bias [44,45]. A further possibility is to superimpose digital models on stable teeth, although they may be subjected to periodontal traction as well as anchorage stress, which makes this method highly unreliable when complex treatment mechanics are used to move teeth with a great root surface area, such as molars. 

In order to evaluate dental movement predictability, ideal post-treatment STL files, exported from the planning software, need to be compared with the pre-treatment digital model. Pre-treatment and post-treatment digital models may include the palatal area, but the virtual plan STL file does not. Thus, another superimposition method from Grünheid et al. [41] was adapted, basing the initial registration on the mesial-buccal cusps of the first molars and the mesial-incisal point of the right central incisor, which was then refined by 50 iterations of a closest-point algorithm to achieve a global best-fit. This method has recently been used by other authors [46,47] and shows excellent accuracy for linear measurements, as well as excellent reproducibility [43]. Differently, in our study soft tissue areas were included when the final global best-fit was performed.

The risk inherently involved in identifying and manually placing landmarks at the cusp tips was addressed in two different ways. Firstly, a protocol including molar segmentation and a surface-based best fit of the occlusal surface of the specific molar ensured that every point chosen was the same on both digital models, thus highly reducing the variability of landmark identification when comparing two digital models. Then, the ICC was used to assess the reproducibility of the measurements and showed excellent agreement for both intra-examiner (0.995) and inter-examiner (0.986) reliability.

A cutoff of 0.5 mm for linear measurements and 2° for angular measurements was introduced to account for model superimposition error; values below cutoffs were considered clinically insignificant and could falsely increase the accuracy, as small movements usually perform very well.

Several studies analyzed upper molar distalization with methods different from digital model superimposition, including cephalometric radiographs and finite element model analysis [27,48,49]. 

According to a finite element analysis conducted by Rossini et al. [50], attachments are mandatory for controlling the bodily movement of the upper second molar; moreover, they indicated a configuration with rectangular vertical from canine to second molar as the most promising. This is consistent with previous studies [27,49] based on cephalometric radiographs, in which attachments from canine to second molar are reported to increase the quantity of distalization and to be effective in controlling bodily movement, without significant distal tipping. Moreover, Garino et al. [49] highlighted that the presence of attachments not only impacts the distalization phase, but also plays a relevant role in the anterior retraction phase by maximizing posterior anchorage, which results in higher values of anterior retraction and less distal tipping of the incisors.

Ayidağa et al. [40] recently compared the effects of maxillary molar distalization among three different groups in a nonlinear finite element study. The first group had vertical rectangular attachments, the second group had horizontal attachments, named as “guideline attachments”, and the third had no attachments. The results suggested that the group without attachments experienced uncontrolled tipping, while the attachment groups showed at least some distal movement of the roots. In addition, the horizontal-attachment group showed higher values of radicular distal displacement and more homogenous distribution of stress through the length of the root than the vertical-attachment group, indicating better control of the radicular movement. However, crown distal displacement was always higher than root distal displacement, suggesting that some tipping occurred anyway. Consistent with most recent evidence, patients in our study were treated with a standardized distalization protocol, including horizontal attachments on molars, in order to achieve bodily distal movement, although measuring the amount of tipping was not a purpose of this study.

Maxillary first molars are frequently rotated around a pivotal axis passing through the mesio-palatal cusp [51]. In cases of mild Class II molar relationship from the buccal side, which are often Class I from the lingual side, correcting the rotation could be enough to distalize the vestibular cusps and obtain a Class I; in moderate and severe cases of Class II, skeletal or dento-alveolar sagittal correction may be required.

Studies performed with the Invisalign^®^ aligner system showed similar results. Indeed, in a prospective study [47], the accuracy of upper-molar derotation was around 43%, and the rotation of canines, premolars, and molars was generally considered a challenging movement with clear aligners. Recently, Lione et al. [52] reported a predictability of 60% for the rotation of upper first molars in growing patients with edge-to-edge Class II before the eruption of the second molars; with a mean derotation of 6°, around 1 mm of arch space was gained. In our study, the accuracy of derotation was 77.5% for the first molars and 62.7% for the second molars, which indicates a moderate to high predictability when a mean derotation of 11.6° is planned. These values appear higher than the ones previously reported. A possible reason is that the distalization protocol may improve the biomechanics of the system, thus increasing the predictability of upper-molar derotation. The second molar, being the tooth that lies furthest back in the arch and having a short tooth crown, is likely to derotate less.

This study presents some limitations: The sample size might be considered small and includes only adult patients older than 18 years old, so no data are present on adolescents or children. However, the study was prospective, and the a priori sample size calculation supports the achieved sample size. Furthermore, it was not possible to assess the compliance of patient in wearing aligners or elastics, nor the possible effect of elastics on the amount of distalization. In an attempt to reduce possible bias due to elastics, all cases included in this study were treated with a 50% distalization protocol with the need for Class II elastics all day. The use of a 50% distalization protocol was chosen considering the lower need for anchorage during distalization, with respect to a 25% distalization protocol. Obviously, considering that the study was conducted in a clinical setting, if an overcorrection was observed doctor asked patients to wear elastics only during the night, or stop wearing them. Further limitations are related to the manual landmark identification by the examiner, but the ICC data support the reliability of the method. Finally, this study focused on only one aligner typology; further studies are needed to compare different distalization protocols and different aligner brands. However, considering the great availability of aligner brands in the market, it is also important to assess the clinical performance of other aligner systems.

## 5. Conclusions

Despite being traditionally regarded as a mechanically challenging treatment option, the outcomes of this study indicate that maxillary molar distalization, measured at the buccal cusp tips, and molar derotation with clear aligners are effective, although the clinician’s prescription, which is the ideal end-treatment goal, is not likely to be fulfilled. Therefore, refinements are necessary.

Overall, the accuracy of buccal cusp distalization was 69.3% for the first molar and 75.2% for the second molar, with a mean prescription of 2 mm. The mesio-buccal cusp of the second molar showed the greatest mean accuracy of the present study, with 79.9%.

Molar derotation reached a mean accuracy of 77.5% and 62.7% for first and second molars, respectively.

## Figures and Tables

**Table 1:** Descriptive statistics of Prescription and Achieved Movement.

			Prescription				Achieved Movement				|AM-P|	P v AM
	Cusp	n	Mean	SD	CI 95% LL	CI 95% UL	Mean	SD	CI 95% LL	CI 95% UL	Mean ± SD	*p* Value
M1 Distalization (mm)	MB	26	1.82	0.90	1.45	2.18	1.30	0.88	0.95	1.66	0.51 ± 0.56	<0.001
	DB	27	1.97	0.92	1.60	2.33	1.42	0.94	1.04	1.79	0.53 ± 0.60	<0.001 *
M2 Distalization (mm)	MB	20	2.25	1.01	1.77	2.72	1.76	1.14	1.22	2.29	0.49 ± 0.64	0.0028
	DB	22	2.13	1.10	1.64	2.62	1.54	1.13	1.03	2.04	0.60 ± 0.65	<0.001
M1 Derotation (°)		29	11.77	5.97	9.50	14.04	8.09	4.80	6.26	9.91	3.68 ± 4.67	<0.001
M2 Derotation (°)		26	11.28	7.03	8.44	14.12	6.40	4.14	4.73	8.07	4.89 ± 4.92	<0.001 *

* Wilcoxon signed-rank test. M1, maxillary first molar; M2, maxillary second molar; mm, millimeters; °, degrees; MB, mesio-buccal cusp; DB, disto-buccal cusp; CI 95% LL, confidence interval 95% lower limit; CI 95% UL, confidence interval 95% upper limit; |AM-P|, absolute value of the mean difference between Achieved Movement and Prescription.

**Table 2:** Descriptive statistics of Accuracy for distalization and derotation.

	M1 Accuracy (%)				M2 Accuracy (%)				M1 v M2
	Mean	SD	CI 95% LL	CI 95% UL	Mean	SD	CI 95% LL	CI 95% UL	*p* Value
Distalization/MB	67.96	30.56	55.62	80.30	79.89	35.25	63.39	96.38	>0.05
Distalization/DB	70.67	29.34	59.06	82.27	70.47	37.20	53.98	86.96	>0.05
Derotation (°)	77.54	41.21	61.87	93.22	62.66	33.57	49.10	76.22	>0.05

M1, maxillary first molar; M2, maxillary second molar; MB, mesio-buccal cusp; DB, disto-buccal cusp; CI 95% LL, confidence interval 95% lower limit; CI 95% UL, confidence interval 95% upper limit.

## Data Availability

The data presented in this study are available on request from the corresponding author.
